# Measuring quality of life impairment in skeletal muscle channelopathies

**DOI:** 10.1111/j.1468-1331.2012.03751.x

**Published:** 2012-05-19

**Authors:** V A Sansone, C Ricci, M Montanari, G Apolone, M Rose, G Meola, INQoL Group

**Affiliations:** a Department of Neurology, University of Milan, IRCCS Policlinico San DonatoMilan, Italy; b Clinical Epidemiology and Biometry Unit, IRCCS Policlinico San DonatoMilan, Italy; c Mario Negri Institute, Department of Oncology, Laboratory for Translational and Outcome ResearchMilan, Italy; d Department of Neurology, Kings CollegeLondon, UK

**Keywords:** INQoL, myotonic dystrophy, non-dystrophic myotonias, quality of life, SF-36, skeletal muscle channelopathies

## Abstract

**Background and purpose:**

Fatigue and pain have been previously shown to be important determinants for decreasing quality of life (QoL) in one report in patients with non-dystrophic myotonia. The aims of our study were to assess QoL in skeletal muscle channelopathies (SMC) using INQoL (individualized QoL) and SF-36 questionnaires.

**Methods:**

We administered INQoL and SF-36 to 66 Italian patients with SMC (26: periodic paralysis, 36: myotonia congenita and 4: Andersen-Tawil) and compared the results in 422 patients with myotonic dystrophies (DM1: 382; and DM2: 40).

**Results:**

(i) INQoL index in SMC is similar to that in DMs (*P* = 0.79). (ii) Patients with myotonia congenita have the worst perception of QoL. (iii) Myotonia has the most detrimental effect on patients with myotonia congenita, followed by patients with DM2 and then by patients with DM1 and hyperkalemic periodic paralysis. (iv) Pain is a significant complaint in patients with myotonia congenita, hypokalemic periodic paralysis and DM2 but not in DM1. (v) Fatigue has a similar detrimental effect on all patient groups except for patients with hyperkalemic periodic paralysis in whom muscle weakness and myotonia more than fatigue affect QoL perception. (vi) Muscle symptoms considered in INQoL correlate with physical symptoms assessed by SF-36 (R from −0.34 to −0.76).

**Conclusions:**

QoL perception in patients with SMC is similar to that of patients with DMs, chronic multisystem disabling conditions. Our results provide information to target treatment and health care of these patients. The sensitivity of INQoL to changes in QoL in the SMC needs to be further explored in longitudinal studies.

## Introduction

Skeletal muscle channelopathies (SMC) are a heterogeneous group of rare muscle disorders having in common muscle weakness and myotonia [1–3]. In general, these are considered ‘benign’ disorders because weakness is episodic, and the heart and lungs are usually unaffected. Mainly for this reason and for the rarity of these disorders, studies on the impact of muscle symptoms on these patients' perception of quality of life (QoL) have been limited. The only detailed study in a group of genetically determined non-dystrophic myotonias using the generic health measure SF-36 demonstrated that painful myotonia and fatigue significantly affect health status in these patients [4].

Determining which symptom or domain most affects health status in patients with SMC is important because this can direct treatment strategies and improve health management of these patients. This is especially true because at the moment, no drug is approved for the treatment of the periodic paralysis and only 40% of patients generally receive treatment. A previous study [5] demonstrated the superiority of dichlorophenamide over acetazolamide in the treatment of persistent interattack weakness in hypokalemic periodic paralysis. This observation prompted a randomized trial of dichlorphenamide (DCP) in the periodic paralyses [6]. In this study, when subjects were asked in a blinded fashion what treatment they preferred, most of them, either hypokalemic or hyperkalemic subjects, preferred DCP over their baseline medication. However, QoL was not addressed in these trials, and the impact of treatment on the patient's QoL or perception of QoL was not explored.

A recent double-blind, placebo-controlled crossover trial has shown that mexiletine is an effective antimyotonia treatment in DM1 [7]. Unfortunately, QoL was not assessed in this study. Also, there is a lack of high-quality randomized evidence in the treatment of myotonia in the non-dystrophic myotonias.

Health status can be measured using QoL questionnaires. These can be generic, like SF-36, or symptom specific, like individualized (IN) QoL. In particular, INQoL is so far the only validated questionnaire that specifically refers to myotonia as one of the common muscle symptoms affecting particular areas of an individual's life [8,9]. QoL questionnaires can be used in everyday clinical practice to assess whether any improvement in muscle strength or myotonia, for instance, has an impact on QoL. This could be regarded as compelling evidence that the intervention is worthwhile. If it can be demonstrated that the tested drug or treatment can improve QoL, then QoL questionnaires could be included as outcome measures in clinical trials.

The general aims of our study were to measure QoL in SMC using the INQoL and SF-36, and to determine the impact of these disorders, as a group, on the patients' health status. More specific aims were to see whether we could identify any difference in QoL perception amongst the different subgroups of channelopathies and, finally, to determine which was the symptom having the most detrimental effect on each subgroup.

## Methods

### Patients

Sixty-six patients with clinically and genetically defined SMC (25 women and 41 men; mean total age: 43.0 ± 14.0 years) were studied. Of these, 16 met diagnostic criteria for hypokalemic periodic paralysis type 1 (CACN: R528M, *n* = 9; R1239H, *n* = 7); 36 for autosomal recessive myotonia congenita (ClCN: A531V, *n* = 11; F167L, *n* = 12; Q812X, *n* = 2; R105C, *n* = 8; G190S, *n* = 3); 10 for hyperkalemic periodic paralysis (SCN4: I592V, *n* = 6; I448G, *n* = 3; T704M, *n* = 1) and four for Andersen-Tawil Syndrome (Kir 2.1: R218Q on KCNJ2 gene). All patients were ambulatory. None had cardiac or respiratory disease. Nineteen patients were on treatment with either DCP (50 mg bid) or acetazolamide (125 mg bid) for periodic paralysis or with mexiletine (200 mg tid) for myotonia (33%).

Results were compared with 422 age- and disease duration-matched, moderately-affected, ambulatory patients with myotonic dystrophy type 1 (DM1) and type 2 (DM2) (228 women and 194 men). Of these, 382 were DM1, with CTG repeats between 600 and 800, and 40 were genetically confirmed DM2 patients. Myotonic dystrophies were chosen as the control group because they are a disabling chronic disease, having in common symptoms like muscle weakness and myotonia, typically also present in the non-dystrophic myotonias. Thirty-four patients with DM1 were on treatment with mexiletine (200 mg tid) for myotonia (11%).

Patients with SMC and DM were matched for age (43.0 ± 14.0 vs. 46.3 ± 14.0, *P* = 0.03) and disease duration (26.5 ± 16.0 vs. 17.2 ± 10.1, *P* = 0.02).

To rule out that QoL perception could be affected by cognitive abnormalities, MMSE was performed in these patients. Patients with MMSE scores below normal range corrected for age and education (< 20) were excluded from the study.

Participants were included in the study as part of a multicenter UILDM-Telethon 3-year grant given to VS (from 2006 to 2009). The research plan was to validate INQoL in Italy in several muscle diseases, including the SMC. All patients gave informed consent to respond to both INQoL and SF-36. Approval from each Ethic Board Committee was obtained at each site (Telethon grant given to VS GUP05001).

### Neuromuscular assessment

Muscle strength was measured according to the modified 0–5 MRC scale in all patients. Myotonia was quantified using an arbitrary four-point self-assessment scale (from 0 = no myotonia to 3 = severe myotonia) in five different body parts (eyes, tongue, jaw muscles, hands and lower limbs).This four-point score was given by the patients for each body part listed, but only the max score was used to quantify myotonia, irrespective of which body part it referred to. Body parts are listed in this subjective scale to facilitate the patients in thinking of their symptoms.

### QoL measures

A symptom-specific QoL questionnaire, called the Individualized Neuromuscular Quality of Life (INQoL) [8], was recently validated in Italy in more than 1000 patients with different muscle disorders having in common progression of muscle symptoms and disability [9]. INQoL consists of 45 questions within 10 sections. Four of these refer to the impact of common muscle disease symptoms like weakness, myotonia (locking), pain and fatigue. Five look at the degree and importance of impact of the muscle disease on particular areas of life (activities, independence, relationships, emotions and body image). The last section looks at treatment and its effects and expectations. Symptoms and impact of these are referred to as the perception of the disease, in general, with no reference to a specific time frame in the INQoL (‘at the moment’). Participants respond using a seven-point Likert scale giving their view of the degree of impact of a symptom or the degree of impact of muscle disease on an aspect of their life together with the importance that they attach to each item, thus allowing a patient-weighted score to be given for each section. The final score from each section is presented as a percentage of the maximum detrimental impact with a higher percentage, indicating greater symptom impact or worse QoL. A composite score can also be obtained from five preselected sections (scales) assessing the impact of the muscle disease on particular areas of life, this representing overall QoL. The higher the INQoL index, the worst is the perception of the patients' QoL.

In the validation process in Italy [9], INQoL demonstrated good reliability, internal consistency and pyschometric validity. No significant correlation was found between cognitive and demographic parameters. Concurrent validity between INQoL index and SF-36 scales showed an association between physical (*P* < 0.0001) more than mental health index (*P* = 0.0092). The results of this survey suggested that the questions in the INQoL are more relevant to those with muscle disease than a generic questionnaire and more sensitive to the life changes that occur as the muscle condition progresses. For these reasons, patients were asked to fill in INQoL and SF-36.

### Statistical analysis

Location and dispersion indexes of continuous variables (mean, median, first and third quartiles and standard deviation) were used to describe the sample (Table 1). Non-continuous variables were described by percentages.

**Table 1:** INQOL and SF-36 subscale score comparisons between skeletal muscle channelopathies (SMC) and myotonic dystrophies (DM). In grey the symptom/domain which most affects QoL. Data reported as median (interquartile range) or as mean ± standard deviation if normally distributed

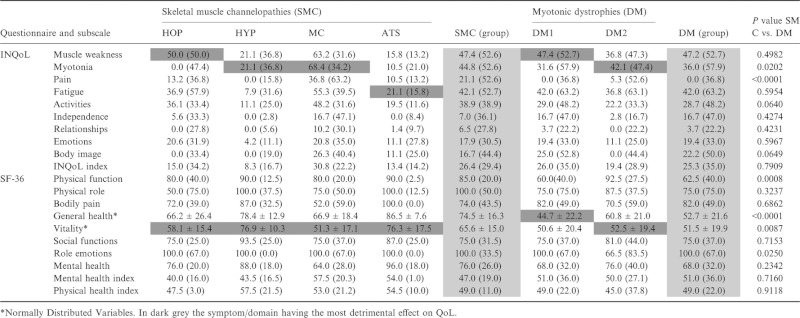

We performed unpaired comparison using parametric *t*-test or Mann–Whitney *U*-test according to variable skewness when looking at the different subgroup analysis.

All statistical evaluations were performed by SAS software package vers.9.1.2., http://www.sas.com/corporate/index.html; the α value of 0.05 was considered statistically significant, and all statistical tests were two tailed.

## Results

INQoL index in SCM considered as a group is similar to that in DM (median 26.4 in SCM vs. 25.3 in DM, *P* = 0.79) (Table 1). Physical health index summary (49 in SCM vs. 49 *P* = 0.91) and mental health index summary (47 in SCM vs. 51 in DM, *P* = 0.71) as measured by SF-36 are also similar in both groups of patients when considered as a whole. Amongst the different subgroups of patients with SMC, patients with myotonia congenita are those having the worst QoL perception (30.8), followed by patients with hypokalemic periodic paralysis HOP (15.0), Andersen-Tawil ATS (13.4) and hyperkalemic periodic paralysis HYP (8.3).

When looking at the different symptoms/domains affecting QoL perception amongst the SMC and the myotonic dystrophy patients in general, there is no significant difference except for the domains ‘myotonia’ (*P* = 0.02) and muscle-related ‘pain’ (*P* < 0.0001), which are worst in SCM (Table 1).

Muscle weakness is detrimental in QoL perception especially in patients with myotonia congenita (63.2), hypokalemic periodic paralysis (50.0) as it is in patients with myotonic dystrophies and especially type 1 (47.4, *P* = 0.49).

Myotonia is the symptom that has the highest impact on patients with myotonia congenita (68.4), followed by patients with myotonic dystrophy type 2 (42.1), myotonic dystrophy type 1 (31.6) and then by patients with hyperkalemic periodic paralysis (21.1) with a significant difference between these groups (MC vs. DM2, *P* = 0.02 and MC vs. DM1 *P* < 0.001; HYP vs. DM2, *P* = 0.01). This is in contrast to findings in the DM1 population in whom muscle weakness (47.4) and fatigue (42.0) rather than myotonia (31.6) are ranked as the symptoms having the highest impact.

Muscle pain seems to be a complaint only amongst patients with MC (36.8) amongst the SMC with a significant difference compared with the myotonic dystrophies where only patients with DM2 complain of muscle pain (5.3, *P* < 0.0001).

Fatigue has a similar detrimental effect on all patient groups (*P* = 0.59) except for patients with hyperkalemic periodic paralysis in whom muscle weakness and myotonia rather than fatigue most affect their QoL perception.

When comparing INQoL with SF-36, weakness considered in INQoL significantly correlates with physical role and function assessed by SF-36 (*R* −0.44 for physical role and −0.67 for physical function); myotonia only considered in INQoL shows a weaker correlation with physical role (*R* = −0.34) and physical function (*R* = −0.47). When considering pain, there is a strong correlation with bodily pain assessed by SF-36 (*R* = −0.76); the correlation is weaker between pain as determined by INQoL and physical role and function (*R* = 0.37 and −0.35, respectively). Fatigue significantly correlates with physical role and function (*R* = −0.49 and −0.65, respectively).

INQoL domain ‘activities’ strongly correlates with SF-36 subscore ‘physical function’ (*R* = −0.72) and more weakly correlates with ‘social function’ (*R* = −0.54). A moderate correlation is observed between the domains ‘relationships’ and ‘emotions’ with ‘social activities’ and ‘role emotion’ and ‘mental health’ assessed by SF-36 (*R* = −0.40, −0.52, −0.38, respectively). INQoL index and SF-36 mental health index MHI (*R* = −0.30) show a weaker correlation than INQoL index and SF-36 physical health index PHI (*R* = −0.44), (Table 2).

**Table 2:** Spearman correlation estimates and 95% confidence limits between INQoL and SF-36 subscales

INQoL Subscale	SF-36 Subscale	*R*-Spearman [95% Cl]	*P* value
Weakness	Physical role	−0.44 [−0.60; −0.25]	< 0.0001
	Physical function	−0.67 [−0.78; −0.53]	< 0.0001
Myotonia	Physical role	−0.34 [−0.51; −0.13]	0.0019
	Physical function	−0.47 [−0.62; −0.27]	< 0.0001
Pain	Bodily pain	−0.76 [−0.84; −0.65]	< 0.0001
	Physical role	−0.37 [−0.53; −0.16]	< 0.0001
	Physical function	−0.35 [−0.53; −0.14]	0.0008
Fatigue	Physical role	−0.49 [−0.64; −0.31]	< 0.0001
	Physical function	−0.65 [−0.76; −0.51]	< 0.0001
Activities	Physical function	−0.72 [−0.81; −0.59]	< 0.0001
	Social function	−0.54 [−0.67; −0.36]	< 0.0001
Relationships	Social activities	−0.40 [−0.57; −0.20]	0.0002
Emotions	Role emotions	−0.52 [−0.66; −0.34]	< 0.0001
	Mental health	−0.38 [−0.57; −0.15]	0.0013
INQol index	Mental health index (MHI)	−0.30 [−0.57; −0.15]	0.0134
	Physical health index (PHI)	−0.44 [−0.61; −0.21]	0.0002

When looking at the individual scores on the subscales included in the SF-36, vitality is considered by all patients with channelopathies as being the domain that is most affected by the disease, with a significant difference (*P* = 0.009) in favour of patients with channelopathies compared with myotonic dystrophies.

Within the channelopathies subgroups, MC is the subgroup in whom vitality is most affected, especially when compared with HYP and ATS. A statistical significant difference was found when comparing MC vs. HYP and ATS (MC vs. HYP *P* = 0.003; MC vs. ATS, *P* = 0.033). No significant difference was found between MC and HOP (*P* = 0.65). No significant difference was also found for HYP vs. HOP (*P* = 0.05), HYP vs. ATS (*P* = 0.99) and HOP vs. ATS (*P* = 0.18). In addition, patients with SMC perceive that their general health is significantly better (*P* < 0.0001) compared with patients with DM.

Weakness and myotonia INQoL subscales positively correlate with disease duration [weakness *R* = 0.30 (0.19; 0.40); *P* < 0.0001; myotonia *R* = 0.20 (0.09; 0.31), *P* = 0.0003].

Age significantly correlates with weakness and pain but not with myotonia [weakness *R* = 0.21 (0.12; 0.30); *P* < 0.0001; pain *R* = 0.12 (0.03; 0.21); *P* = 0.018; myotonia *R* = 0.02 (−0.08; 0.11), *P* = 0.71] in all groups of patients.

A statistically significant correlation was found between subjective perception of weakness measured by INQoL weakness subscale and measures of muscle strength (MMRC) for all groups of patients [muscle weakness vs. MMRC: *R* = −0.33 (−0.53; −0.09), *P* = 0.008]. A strong and statistically significant correlation was found between the four-point Likert subjective scores of severity of myotonia with muscle weakness and myotonia INQol subscales in patients with MC and HYP [weakness *R* = 0.75 (0.50; 0.88); *P* < 0.0001; myotonia *R* = 0.65 (0.34; 0.83), *P* = 0.0002].

When looking at the effects of sex on QoL perception, women from both group of patients have a similar perception to men except for the INQoL domain ‘emotions’ in which women have a worse emotional perception compared with men (*P* = 0.006).

## Conclusions

QoL is impaired in patients with SMC, and it is perceived as detrimental as it is by patients with DM, suggesting that the term ‘benign’ potentially applicable to SMC needs to be reconsidered.

In fact, a previous report on QoL in the non-dystrophic myotonias [4] demonstrated that QoL is significantly impaired in patients with sodium and chloride channelopathies. Painful myotonia and fatigue closely correlate with low-health status in these patients [4]. Interestingly, our data seem to confirm these findings. Patients with MC are the ones having, in general, the worst perception of QoL, and myotonia proves to be most disabling symptom. Myotonia is the most disabling symptom also in our patients with HYP and DM2. In agreement with clinical experience, despite the fact all patients with DM1 had clinical myotonia, this was not perceived as the most detrimental domain affecting QoL. This suggests that myotonia should be the primary target of treatment in patients with MC, HYP and DM2, and improvement of myotonia should be the primary outcome measure during a treatment trial in patients with MC, HYP and DM2.

In addition to myotonia, muscle weakness and fatigue impair QoL perception in patients with MC more than these symptoms affect other subgroups of patients. Again, this is in agreement with the previous report by Trip and co-workers 2009 where fatigue was the strongest predictor of low general health perception in patients with sodium and chloride channelopathies. A permanent myopathy is less frequent in patients with hyperkalemic periodic paralysis [10–12] than in those with hypokalemic periodic paralysis [5,12–14], and episodes of weakness are often shorter in duration so that these two factors may justify a better perception of QoL in HYP patients compared with MC and HOP patients. Fatigue is the domain that most affects QoL in ATS, and how much this is related to the muscle condition or the cardiac impairment needs to be further explored [15].

Similarly, in agreement with previous reports [4,16], pain was a complaint in myotonia congenita and in DM2, again suggesting that future treatment trials may also consider pain as an outcome measure. In this respect, to better interpret the impact of myotonia and of pain on QoL perception, quantification of both myotonia and pain are recommended when interpreting INQoL results in any potential treatment trials.

General health and vitality are in general perceived as being better in the SMC compared with DM. This may be due to the multisystem involvement of DM where impairment of the heart, sleep, breathing and sight may have an additional negative impact on these patients’ perception of QoL.

Skeletal muscle channelopathies do not in general involve the brain and even though visual spatial impairment has been described in patients with ATS [17], the degree, impact and extent to which this is related to the muscle condition and affects QoL perception needs to be further explored. Patients with SMC have a similar detrimental perception of QoL as patients with DMs. In fact, it could have been expected that, despite having excluded patients with abnormal MMSE, the disexecutive syndrome nonetheless described in patients with DM1 [18,19] might have influenced QoL perception in these patients differently from that of patients with SMC in whom frontal lobe involvement is not a typical feature.

The stronger correlation between INQoL index and SF-36 physical more than mental health index suggests that INQoL may be able to capture physical limitations owing to the muscle condition. In addition, INQoL, and not SF-36, provides information on myotonia and the extent by which this has a detrimental effect on QoL perception. This allows to pick out differences amongst the channelopathies that are not captured by SF-36 alone.

Our results have been obtained in a rather large sample of patients with SCM, given the rarity of these disorders. However, the numbers in the subgroups are still small and results will need to be confirmed in larger samples of patients if possible. Sample size may also affect variable skewness. This could be due to the fact that subjects tend to respond to questions regarding most domains with the most detrimental score or with the least detrimental score (i.e. most patients report they have either pain or no pain creating asymmetry in value distribution). Vitality and general health describe more general aspects of patients’ QoL perception so that responses tend to distribute symmetrically around a mean value.

Our results allow us to draw some preliminary conclusions. First, INQoL allows measurement of QoL in SMC. Whether INQoL will be able to capture sensitivity to change in the skeletal channelopathies will have to be further explored, but our data demonstrate that it can quantify the impact of muscle symptoms that are specific to this group of patients (e.g. myotonia, muscle pain). Therefore, we recommend that this instrument be used in combination with more generic health measures to capture information that may be more relevant to these muscle diseases. Knowing which symptom affects QoL allows targeting treatment and health care needs of these patients. Therefore, improvement in QoL as assessed by QoL questionnaires like the INQoL should be included as outcome measures in potential treatment trials. Secondly, MC patients seem to be the subgroup of patients with the worst perception of QoL. It may be necessary to consider these patients as a separate group in any potential treatment trial to better address these patients' needs. Thirdly, as already pointed out, myotonia should be the treatment target for patients with MC, HYP and DM2, and improvement of myotonia should be the primary outcome measure in therapeutic trials including these patients.

## Appendix

INQoL group members

Panzeri M: Department of Neurology, IRCCS Policlinico San Donato, University of Milan; Angelini C, Palmieri A: Department of Neurosciences, University of Padua; Siciliano G, Volpi L, Falorni M: Department of Neurosciences, University of Pisa; Mongini T, Vercelli L: Department of Neurosciences, University of Turin; Politano L, Tozza S, Solimene C: Department of Cardiomyology and Clinical Genetic, University of Naples; Massa R, Panico MB, Pisani V: Department of Neurosciences, University of Tor Vergata, Rome; Grandi M: Respiratory Physiopathology, Costamasnaga, Como; Toscano A, Musumeci O, Rodolico C: Neurological and Neurosurgery Institute, University of Messina.
